# Racial/Ethnic Differences in Glycemic Control in Older Adults with Type 2 Diabetes: United States 2003–2014

**DOI:** 10.3390/ijerph17030950

**Published:** 2020-02-04

**Authors:** Brittany L. Smalls, Tiarney D. Ritchwood, Kinfe G. Bishu, Leonard E. Egede

**Affiliations:** 1 Department of Family and Community Medicine, University of Kentucky College of Medicine, 2195 Harrodsburg Road, Suite 125, Lexington, KY 40505, USA; 2 Department of Family Medicine and Community Health, Duke University School of Medicine, 2200 West Main Street, Suite 600, Durham, NC 27705, USA; tiarney.ritchwood@duke.edu; 3 College of Medicine, Medical University of South Carolina, 135 Rutledge Ave, Charleston, SC 29425, USA; bishu@musc.edu; 4 Center for Advancing Population Science, Medical College of Wisconsin, 9200 W. Wisconsin Ave, Milwaukee, WI 53226, USA; legede@mcw.edu

**Keywords:** older adults, diabetes, race/ethnicity, glycemic control, NHANES

## Abstract

The aim of this study was to determine whether racial differences in HbA1c persist in older adults (≥65 years) living with type 2 diabetes. Data from The National Health and Nutrition Examination Survey (NHANES) 2003–2014 were used to examine the association between HbA1c and older adults (≥65 years) over time. Compared to non-Hispanic Whites, Mexican Americans had the greatest difference in average HbA1c among minority groups, followed by those with unspecified/mixed ethnicities and non-Hispanic Blacks. In the adjusted linear model, racial minorities had a statistically significant relationship with HbA1c. There was no relationship between HbA1c and older age and insulin use. Trends in mean HbA1c over time increased for non-Hispanic Blacks and Mexican Americans and decreased for non-Hispanic Whites. The findings suggest that racial differences in HbA1c persist into older age and compared to non-Hispanic Whites, non-Hispanic Blacks and Mexican Americans are at an increased risk of morbidity, mortality, and disability due to high HbA1c. Furthermore, alternate measures of glycemic control may be needed to screen and manage T2DM in racial minorities.

## 1. Introduction

In the United States (U.S.), 30.3 million people or 9.4% of the population were diagnosed with diabetes in 2015, with the vast majority (approximately 95%) suffering from type 2 diabetes mellitus (T2DM) [1]. By 2050, it is estimated that 48 million Americans will be living with T2DM, with 30% of those diagnosed expected to be between 65 and 74 years of age and 33% expected to be 75 years or older [2]. These statistics are particularly concerning, as T2DM is among the top 10 leading causes of death and disability within the United States [3]. Taken together, the current prevalence and projected incidence rates of T2DM are alarming and have the potential to exacerbate existing disparities in health outcomes among members of vulnerable populations, including racial and ethnic minorities and older adults (≥65 years).

While previous research has suggested that T2DM is becoming more prevalent across all strata within the U.S. [4], there have been a number of studies that have linked racial and ethnic background to T2DM-related health disparities [5,6]. Previous research, for example, has shown that African Americans, Hispanics, and American Indians/Alaska Natives are disproportionately impacted by T2DM when compared to non-Hispanic Whites [1,4,7,8]. Specifically, African Americans and Mexican Americans experience more rapid increases in T2DM prevalence and its related complications than their non-Hispanic White peers. Moreover, considering the changing demographics of the U.S., concern is greatest for older adults, as they are living longer and are more likely than their younger peers to suffer from various comorbidities that lead to greater disease burden, healthcare costs, and caregiving needs [9,10]. 

Hemoglobin A1c (HbA1c) has been widely accepted as an indicator of quality T2DM management, severity of disease, and a measure of one’s risk for developing T2DM-related complications. In 2010, the American Diabetes Association suggested that an HbA1c of 6.5% or greater be used to diagnose T2DM [11]. For many years, researchers have acknowledged that HbA1c varies by race and ethnicity; however, these differences have been largely attributed to issues with access to healthcare or healthcare quality [12]. One study using 1999–2000 data from The National Health and Nutrition Examination Survey (NHANES)—a representative sample of the U.S. civilian non-institutionalized population—found that African Americans and Hispanics with T2DM (both diagnosed and undiagnosed) reported higher HbA1c levels than their White peers (8.1%, 8.2%, and 7.6%, respectively) [13]. Findings from additional studies using a variety of national and regional datasets have indicated that, even after controlling for several behavioral (e.g., medication adherence, self-management activities), demographic (e.g., age, sex, education level, marital status), and structural variables (e.g., healthcare access), race/ethnicity differences in HbA1c persisted [14,15,16,17,18]. In a more recent study using 1999–2010 data from NHANES, researchers found that, among older adults (≥65 years of age) diagnosed with diabetes, non-Hispanic Whites were more likely than their Hispanic peers to attain an HbA1c <7% [19]. Moreover, older Hispanics with undiagnosed T2DM had a higher likelihood of having an HbA1c >7% than older non-Hispanic Whites and African Americans [19]. 

Therefore, the primary objective of the current study was to use NHANES data from 2003–2014 to assess racial and ethnic differences in HbA1c among older adults, a population expected to reach 72 million by 2030 [20]. We hypothesized that glycemic control will be worse in non-Hispanic Blacks compared to non-Hispanic Whites. We further hypothesized that glycemic control will be associated with older age, insulin use, and health status. The secondary objective of this study was to determine if there is a difference in HbA1c over time by race in older adults using NHANES 2003–2014. For this objective, we hypothesized that differences in HbA1c by race will persist over the defined period of time and that non-Hispanic Whites will have the lowest mean HbA1c of all race/ethnicity groups at each time point.

## 2. Materials and Methods

We used NHANES 2003–2014 data, a representative sample of the U.S. civilian non-institutionalized population, for adults ≥65 years in this analysis. The data for NHANES are collected by the National Center for Health Statistics annually to evaluate the health and nutritional status of adults and children in the U.S. over time using interviews/surveys, physical examinations, and laboratory tests. For the current study, the sample population was created by merging the NHANES datasets from 2003 through 2014 using the variable “Doctors told you have diabetes (DIQ010)” as an indicator variable. Then, a subpopulation was created to only include those who were ≥65 years. Descriptive analysis was conducted for all variables expressed as means and 95% confidence intervals, proportions, and frequencies, when appropriate. Prior to analysis, race/ethnicity was dichotomized into 4 categories: (1) non-Hispanic White, (2) non-Hispanic Black, (3) Mexican American, and (4) unspecified/mixed racial background. NHANES panel years were grouped into 3 categories: 2003–2006, 2007–2010, and 2011–2014. Bivariate analysis was then used to estimate the mean glycosylated HbA1c (henceforth referred to as “HbA1c”) by race/ethnicity. Pairwise comparison of marginal linear predictions (“pwcompare”) was then used to assess differences in HbA1c by race/ethnicity in adjusted and unadjusted models. Šidák correction was used to account for multiple comparisons [21] between each race/ethnicity using the “sidak” option within the pairwise comparison function in Stata 14. Lastly, linear regression was used to examine the overall relationship between race/ethnicity and HbA1c as well as determine trends associated with change in mean HbA1c for each race/ethnicity over time. Furthermore, when conducting this statistical analysis, the “svyset” command was used with the appropriate survey weights to account for the complex survey design of NHANES. 

## 3. Results

**Figure 1:**
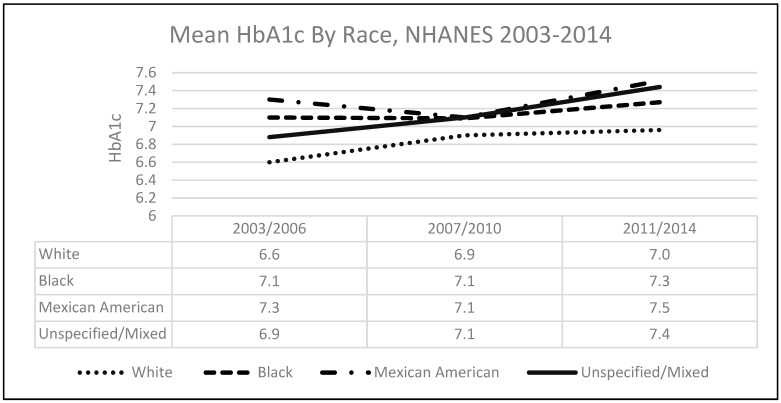
Mean HbA1c by race/ethnicity, NHANES 2003–2014.

The majority of the sample population were women (53.4%), between the ages of 65 and 74 years (59.4%), non-Hispanic Whites (69.3%), college-educated (41.0%), married (58.0%), had an annual income ≥$20,000 (72.8%), a body mass index <30 (51.7%), health insurance (98.3%), described their health as good, very good, or excellent (60.7%), and were not currently using insulin (73%) (see Table 1). Univariate analysis showed mean HbA1c by race/ethnicity are as follows: 6.8% for non-Hispanic White, 7.2% for non-Hispanic Black, 7.3% for Mexican American, and 7.2% for those from unspecified/mixed racial backgrounds. In the unadjusted pairwise comparisons of the marginal linear predictions model, there were statistically significant differences in HbA1c between non-Hispanic Whites and non-Hispanic Blacks (+0.5%; *p* = 0.043) for years 2003 through 2006. There was also a statistically significant difference between non-Hispanic Whites and Mexican Americans from 2003 through 2006 (+0.7%; *p* < 0.001) and a moderately significant relationship from 2011 through 2014 (+0.5%; *p* = 0.053). In the adjusted pairwise comparisons of marginal linear predictions, there was no longer a statistically significant difference in mean HbA1c between non-Hispanic Whites and non-Hispanic Blacks from 2003 to 2006 or non-Hispanic Whites and Mexican Americans between 2011 and 2014 (see Table 2). When considering the overall time period (2003–2014) in the adjusted model, we found that when compared to non-Hispanic Whites, Mexican Americans had the greatest statistically significant difference in HbA1c (+0.4%; *p* = 0.006) whereas unspecified/mixed racial background had a moderately significant difference (+0.4%; *p* = 0.053) (see Figure 1). 

In the unadjusted linear model, compared to non-Hispanic Whites, each race/ethnicity had a statistically significant relationship with HbA1c: non-Hispanic Black (beta = 0.36; *p* < 0.001), Mexican American (beta = 0.44; *p* < 0.001), and unspecified/mixed race (beta = 0.40; *p* < 0.001). In the adjusted model controlling for sex, age, education, annual income, marital status, body mass index, insurance status, health status, and insulin use, the relationship between race/ethnicity and HbA1c weakened but remained statistically significant. Compared to non-Hispanic Whites, each race/ethnicity had a statistically significant relationship with HbA1c: non-Hispanic Black (beta = 0.31; *p* = 0.02), Mexican American (beta = 0.42; *p* = 0.003), and unspecified/mixed race (beta = 0.37; *p* = 0.005). There was also a statistically significant relationship between HbA1c with age category 75–84 years (beta = −0.18; *p* = 0.04) and use of insulin (beta = 0.98; *p* < 0.001) (see Table 3). Next, we assessed the trends in mean HbA1c by race. We found that after accounting for the interaction between each race/ethnicity and panel year, non-Hispanic Blacks (beta = 0.51, *p* = 0.012) and Mexican Americans (beta = 0.62; *p* < 0.001) had a positive overall trend, indicating an increased mean HbA1c over time, whereas unspecified/mixed race did not have a statistically significant trend over time (beta = 0.11, *p* = 0.607). However, non-Hispanic Whites had a statistically significant inverse trend, indicating that over time, mean HbA1c decreased (beta = −0.50, *p* = 0.001).

## 4. Discussion

Among older adults living with T2DM, Mexican Americans, non-Hispanic Blacks, and those with unspecified racial/ethnic backgrounds had higher HbA1c and greater differences in average HbA1c compared to non-Hispanic Whites. Moreover, we found that Mexican Americans living with T2DM who were ≥65 years had poorer glycemic control than non-Hispanic Blacks and those with an unspecified racial/ethnic background. In the adjusted linear model controlling for demographic variables, health status, and insulin use, we found that being Mexican American was the strongest predictor of HbA1c followed by unspecified/mixed race and non-Hispanic Black race/ethnicity when compared to non-Hispanic Whites. Lastly, we found that more insulin use and older age (≥75 years) was associated with higher HbA1c. Taken together, the results of this study suggest that racial and ethnic disparities in HbA1c persist in older age, with Mexican Americans experiencing the greatest challenges when compared to non-Hispanic Whites and unspecified/mixed racial background.

Several factors may explain racial/ethnic differences in HbA1c among older adults. First, as previously indicated, non-Hispanic Blacks, regardless of whether they have T2DM or not, tend to have higher HbA1c levels than their non-Hispanic White peers despite having similar blood glucose concentrations [16,18,22,23,24]. Previous studies have shown that these differences were robust even after controlling for potentially confounding variables, such as age, sex, education, income, and BMI [16,18,22]. Ethnic disparities in HbA1c were even greater for those with prediabetes and clinically diagnosed T2DM [22]. These and similar findings raise concerns about the clinical validity of using HbA1c to monitor T2DM management, particularly among ethnic minorities [24]. Moreover, these findings also raise questions about the validity of using HbA1c alone to screen individuals for T2DM [25], as there is evidence that HbA1c levels overestimate blood glucose concentration among ethnic minorities [24] and that non-Hispanic Blacks may differ in their glycation of hemoglobin [24]. It is also possible that the racial differences identified in this nationally representative sample of older people were due to existing racial differences given that most older adults are diagnosed with T2DM prior to their 65th birthday [26].

The current study is consistent with previous research that identified racial differences in HbA1c between older non-Hispanic Blacks and non-Hispanic Whites [27,28]. It also extends the research in this area, as many of the previous studies had limited racial and ethnic comparisons in HbA1c to include only non-Hispanic Whites and non-Hispanic Blacks. Given the rapidly changing demographics of the U.S., it is critical that research examining health disparities becomes more inclusive. Hispanics represent the largest ethnic minority group within the U.S., and there is much diversity within this group, despite tendencies to develop ethnic clusters for ease of classification. As such, it is crucial that more research be focused on addressing the specific barriers faced among subsets of this population, particularly for older Mexican Americans living with T2DM. Additionally, more research is needed to develop best practices with regard to identifying challenges for those of mixed racial and ethnic background, as all-inclusive categories such as “unspecified/mixed race” limit researchers from identifying specific challenges facing specific individuals with shared cultural and behavioral practices that might contribute to suboptimal HbA1c. This requires that we move beyond simple racial categorizations and consider the influence of ethnic identity and various sociostructural factors.

Additional factors that may contribute to the differences in HbA1c in older adults include social support, lifestyle choices, and multimorbidity. Previous research has suggested that social support [29], particularly a sense of belonging [30], has a significant association with health outcomes in older adults with chronic conditions including T2DM [29,30,31]. Furthermore, social support has been shown to reduce BMI and influence medication adherence in older adults living with T2DM [29,32]. Chiu and Wray (2010) found, in a nationally representative sample, that lifestyle factors (e.g., physical activity, smoking, drinking, and body weight) have a significant relationship with HbA1c [33]. However, older adults are less likely to participate in lifestyle changes, such as physical activity, even though lifestyle interventions to engage older adults in healthy eating and physical activity have been shown to improve HbA1c [34] and reduce complications related to metabolic disorders [35]. Lastly, multimorbidity in older adults can increase disease burden, risk of complications, and distress associated with managing T2DM [36,37].

There are limitations to this study. First, this was a cross-sectional study and did not account for change in HbA1c over time. Next, the analysis did not account for guideline changes in diagnosis of T2DM using HbA1c. Third, we did not account for multimorbidity or geriatric syndromes in our study, which can increase the risk of disease severity, disease burden, and disability [38]. Lastly, there was not enough information to assess the influence of socioenvironmental factors that have been shown to contribute to self-management and HbA1c in those living with T2DM. We could not, for example, account for adherence to T2DM-related self-management (e.g., medication adherence, healthy meal planning, physical activity, blood glucose monitoring) and health literacy by race, which have been previously associated with increased HbA1c [14,39,40].

## 5. Conclusions

Taken together, this study extends previous research by examining racial differences in HbA1c among older adults using a nationally representative sample. While our results indicate that racial minorities have higher HbA1c than non-Hispanic Whites, the causes of such disparities remain unclear. Moreover, adults ≥65 years are all eligible for Medicare and should, in theory, have access to healthcare resources via insurance to manage their chronic conditions, such as T2DM, and achieve optimal health outcomes. This study has shown that, in a nationally representative population, despite access to Medicare, differences in glycemic control persist by race/ethnicity over time for older adults diagnosed with T2DM. Furthermore, historically, older adults have been excluded from health outcomes research or have not been the target population for such research. This study provides a glimpse into the pervasiveness of health disparities for older adults living with T2DM. Additionally, the American Diabetes Association has also indicated that older adults are a vulnerable population, and additional studies are needed to better understand areas to intervene among this patient population [41].

The results of this study inform researchers that challenges to glycemic control among minorities persist into older age and suggest that interventions targeting older adults are needed. Further, research initiatives aimed at addressing disparities not only in non-Hispanic Blacks and Mexican Americans but also those of mixed races/ethnicities are needed. Though individuals who identify as of mixed race/ethnicity are hard to identify, there is an increase the number of people who identify with this category, and it is not in the best interest of science to neglect these individuals when reporting data—any minority status should be taken under consideration when evaluating disparities. Moreover, minority status combined with aging suggests an unsettling prognosis for those living with T2DM. We suggest that future research focus on following older adults over time to better evaluate race/ethnic differences in HbA1c as well as identifying modifiable socioenvironmental factors [42,43,44] that contribute to poor self-management within this population to facilitate optimal HbA1c and better outcomes.

## Figures and Tables

**Table 1:** Study sample demographics, The National Health and Nutrition Examination Survey (NHANES) 2003–2014.

Variable	Percent (Frequency)
**Gender**	
Men	46.6
Women	53.4
**Age**	
65–74 years	59.4
75–84 years	38.4
85 years and up	2.2
**Race/Ethnicity**	
Non-Hispanic White	69.3
Non-Hispanic Black	13.8
Mexican American	5.4
Unspecified/Mixed Race	11
**Education**	
Less than High School	33.7
High School	25.3
College	41
**Annual Income**	
<$20,000	27.2
≥$20,000	72.8
**Marital Status**	
Married	58
Divorced/separated/widowed	39.1
Single	2.9
**Body Mass Index**	
<30	48.3
≥30	51.7
**Covered by Health Insurance**	
No	1.7
Yes	98.3
**General Health Condition**	
Fair or Poor	39.3
Good, Very Good, or Excellent	60.7
**Currently Using Insulin**	
No	73
Yes	27
**NHANES Sample Size by Year**	
2003–2004	14.8 (275)
2005–2006	13.5 (210)
2007–2008	16.0 (337)
2009–2010	17.8 (344)
2011–2012	18.3 (291)
2013–2014	19.6 (310)

**Table 2:** Adjusted pairwise comparisons of marginal linear prediction of hemoglobin A1C by race/ethnicity.

Race/Ethnicity	2003–2006		2007–2010		2011–2014		Overall	
	Diff	*p*	Diff	*p*	Diff	*p*	Diff	*p*
Non-Hispanic Black vs. Non-Hispanic White	0.6	0.078	0.3	0.589	0.2	0.970	0.3	0.073
Mexican American vs. Non-Hispanic White	0.7	0.000	0.2	0.613	0.4	0.625	0.4	0.006
Unspecified/Mixed Race vs. Non-Hispanic White	0.2	0.810	0.2	0.726	0.4	0.173	0.3	0.053
Mexican Americans vs. Non-Hispanic Black	0.1	0.996	−0.1	1.000	0.3	0.914	0.1	0.937
Unspecified/Mixed Race vs. Non-Hispanic Black	−0.3	0.747	−0.0	1.000	0.2	0.919	0.0	1.000
Unspecified/Mixed Race vs. Mexican Americans	−0.5	0.347	0.0	1.000	−0.0	1.000	−0.1	0.964

Note: Controlling for sex, age, education, household income, marital status, body mass index, health status, and insulin use.

**Table 3:** Adjusted linear regression of hemoglobin A1c by race/ethnicity.

Variables	Coefficient	*p*-Value
**Race/Ethnicity**		
Non-Hispanic White *	--	--
Non-Hispanic Black	0.3	0.013
Mexican American	0.43	0.001
Unspecified/Mixed Race	0.32	0.009
**Sex**		
Men *	--	--
Women	−0.06	0.456
**Age**		
65–74 years *	--	--
75–84 years	−0.18	0.026
85 years and older	0.2	0.261
**Education**		
Less than High School *	--	--
High School	−0.01	0.92
College or More	−0.05	0.692
**Household Annual Income**		
Less than $20,000 *	--	--
More than $20,00	0.01	0.911
**Marital Status**		
Married *	--	--
Non-married	0.01	0.843
Never Married	0.06	0.801
**BMI**		
Less than 30 kg/m^2^ *	--	--
More than 30 kg/m^2^	0.01	0.91
**Insurance Status**		
No *	--	--
Yes	−0.35	0.375
**Health Status**		
Fair or Poor *	--	--
Good/Very Good/Excellent	0.01	0.883
**Insulin Use**		
No *	--	--
Yes	0.93	0
**NHANES Year**		
2003–2006 *	--	--
2007–2010	0.14	0.114
2011–2014	0.32	0.001

* Reference group.
